# Current Therapies and Future Horizons in Cardiac Amyloidosis Treatment

**DOI:** 10.1007/s11897-024-00669-7

**Published:** 2024-05-29

**Authors:** Julia Vogel, Alexander Carpinteiro, Peter Luedike, Florian Buehning, Simon Wernhart, Tienush Rassaf, Lars Michel

**Affiliations:** 1 Department of Cardiology and Vascular Medicine, West German Heart and Vascular Center, University Hospital Essen, Hufelandstraße 55, 45147 Essen, Germany; 2 Department of Hematology and Stem Cell Transplantation, West German Cancer Center, University Hospital Essen, Hufelandstraße 55, 45147 Essen, Germany

**Keywords:** Amyloidosis, Heart Failure, Light Chain, Restrictive Cardiomyopathy, Tafamidis, Transthyretin

## Abstract

**Purpose of Review:**

Cardiac amyloidosis (CA) is a condition characterized by misfolding and extracellular deposition of proteins, leading to organ dysfunction. While numerous forms of CA exist, two subtypes dominate clinical prevalence: Transthyretin amyloid (ATTR) and immunoglobulin light chain amyloid.

**Recent Findings:**

The current scientific landscape reflects the urgency to advance therapeutic interventions with over 100 ongoing clinical trials. Heart failure treatment is affected by CA phenotype with poor tolerance of otherwise frequently used medications. Treating comorbidities including atrial fibrillation and valvular disease remains a challenge in CA, driven by technical difficulties and uncertain outcomes. Tafamidis is the first ATTR-stabilizer approved with a rapidly growing rate of clinical use. In parallel, various new therapeutic classes are in late-stage clinical trials including silencers, antibodies and genetic therapy.

**Summary:**

Managing CA is a critical challenge for future heart failure care. This review delineates the current standard-of-care and scientific landscape of CA therapy.

## Introduction

Amyloidosis is characterized by a misfolding of proteins which are deposited extracellularly in organs and tissues, frequently leading to organ dysfunction. Deposition of amyloid fibrils in the heart may lead to severe cardiac insufficiency and cardiomyopathy [1]. In cardiac amyloidosis (CA), two subtypes are responsible in over 90% of all cases: transthyretin amyloid (ATTR) and immunoglobulin light chain amyloid (AL) [2•]. The differentiation between these two forms is paramount as it influences treatment approaches. ATTR-amyloidosis is primarily a result of wild-type (ATTRwt) or mutated (ATTRh) transthyretin protein deposition in the heart, while AL-amyloidosis is associated with the deposition of immunoglobulin light chains [3•]. These subtypes have distinct clinical courses, treatment options, and prognosis, making accurate subtyping a pivotal step in the management of CA.

One of the primary challenges in diagnosing and treating CA lies in its diverse clinical manifestations. The disease may masquerade as various other cardiac disorders, resulting in delayed or missed diagnosis [4]. Symptoms can range from subtle fatigue and dyspnea to overt heart failure (HF), arrhythmias, and sudden cardiac death [5]. The prevalence and incidence of CA is increasing due to improved diagnostics and awareness of treating cardiologists, and studies have shown that TTR-amyloid deposits are the cause of cardiac insufficiency in 13-17% of patients with heart failure with preserved ejection fraction (HFpEF) [6]. Understanding the diverse clinical presentations is crucial for timely and accurate intervention, which can significantly impact patient outcomes [2•].

If left untreated, CA is a fatal disease, emphasizing the need for effective therapies. The increasing recognition of the disease by cardiologists and scientific progress has led to a great dynamic in the development of innovative therapeutic approaches. This article summarizes the current state of science and the outlook for therapies for CA.

## Diagnosis

**Fig. 1:**
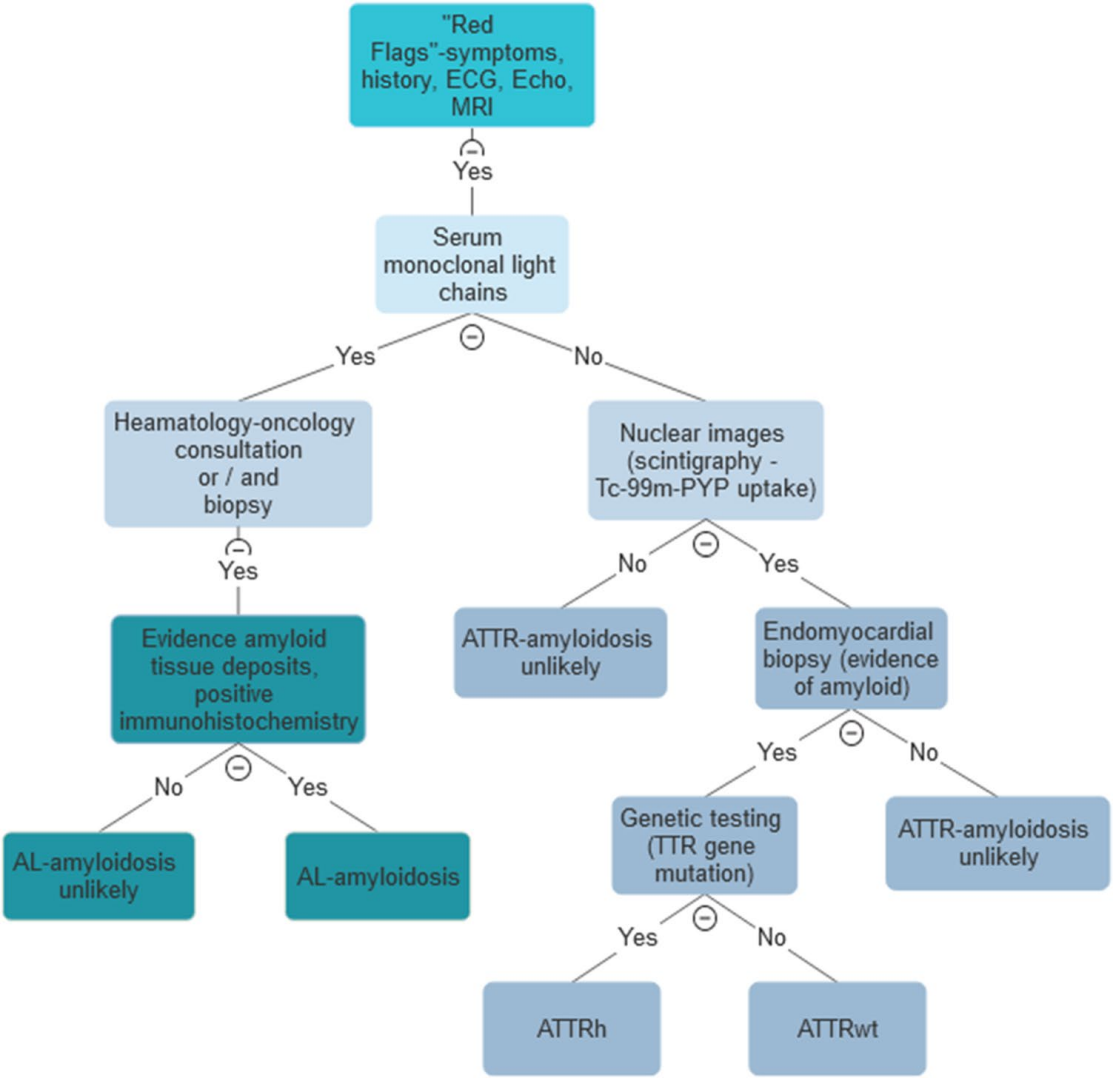
Workflow for the diagnosis of cardiac amyloidosis and the differentiation between ATTR and AL amyloidosis (AL = light chain amyloidosis, ATTR = transthyretin amyloidosis, ATTRh = hereditary ATTR, ATTRwt = wild-type ATTR, ECG=electrocardiography, MRI = magnetic resonance imaging)

Early diagnosis is of crucial importance for the amyloidosis patient. As untreated amyloidosis is a progressive condition, delayed diagnosis leads to advanced stages of the disease. For AL-amyloidosis, a clear correlation has been shown between the time from the appearance of the first symptoms to diagnosis and the prognosis [4]. In ATTR-amyloidosis, starting ATTR-stabilizing treatment at earlier stages of the disease (New York Heart Association (NYHA) I and II) leads to an improvement in prognosis while patients with advanced cardiac involvement (NYHA III) do no longer benefit [7•]. It should be noted that the NYHA classification often reflects a subjective assessment by the patient. Patients with cardiac amyloidosis frequently have comorbidities, making an assessment according to NYHA more challenging. Non-specific symptoms such as breathlessness, peripheral oedema, rhythm disturbances, polyneuropathy, or gastrointestinal complaints can delay the diagnosis. The European Society of Cardiology recommends screening for CA if the left ventricular wall exceeds 12 mm in transthoracic echocardiography (TTE) and if there is suspicion of systemic amyloidosis with presence of at least one *red flag* (aortic stenosis >65years, HF >65years, polyneuropathy, carpal tunnel syndrome, spinal canal stenosis, macroglossia etc.) [2•]. Figure 1 shows a workflow for the diagnosis of CA.

Blood work, electrocardiogram (ECG), and TTE can substantiate the suspected diagnosis. The use of circulating cardiac biomarkers such as troponin and natriuretic peptides is useful, not only for diagnostic, but also for management, staging, and prognostication [8, 9]. Renal function is often compromised in AL-amyloidosis; therefore, renal function, creatinine and serum-albumin levels should be assessed including urinalysis to indicate and specify proteinuria [10]. Urine and serum immunofixation and analysis of free light chains are a crucial diagnostic tool for the diagnosis of AL-amyloidosis [11]. ECG patterns may provide initial clues, with specific changes like low-voltage QRS, or a lack of R wave progression in anterior precordial leads, indicating the condition [12]. Conduction defects like atrio-ventricular (AV) block are more common in ATTR than in AL [13]. However, ECG findings may be late-stage indicators with low sensitivity and specificity [12]. Transthoracic echocardiography is the most used diagnostic tool for diagnosing CA. Key diagnostic clues include increased left ventricular (LV) wall thickness, a non-dilated left ventricle, abnormal myocardial appearance, pericardial effusion and diastolic dysfunction. [3•]. Especially LV wall thickness ≥12mm is the hallmark feature of CA and requires further evaluation [14]. Global longitudinal strain with apical sparing, a sensitive measure of myocardial dysfunction, offers additional diagnostic insights [15] and is one of the most sensitive marker for the presence of CA [14]. The amyloid deposits lead to a "speckled" appearance on imaging and may affect the thickness of the heart walls (15).

Multimodal imaging is used to confirm the final diagnosis. Cardiac magnetic resonance (CMR) is a valuable tool, particularly when ultrasound quality is poor. CMR helps identifying characteristic patterns of late gadolinium enhancement, presumably offering insights into disease progression and prognosis [16] [17]. However, CMR cannot distinguish between ATTR- and AL-amyloidosis or is reliable for classifying [18]. CMR could become increasingly important in the future, as it is already being used to monitor disease progression [19]. Cardiac nuclear scintigraphy, using bone- avid radiotracers, is a crucial method for diagnosing ATTR-amyloidosis, especially when serum and urine tests for AL-amyloidosis yield negative results [20]. Various radiopharmaceuticals can be used, with quantification of radiotracer uptake aiding in diagnosis [16]. A diagnostic algorithm recommends bone scintigraphy for ATTR-amyloidosis diagnosis, followed by genetic testing to distinguish between hereditary and wild-type amyloidosis [10]. Endomyocardial biopsy aids in identifying the subtype of amyloidosis, as tissue typing becomes essential [3•], meaning if CA is highly suspected and imaging is inconclusive, a biopsy should be performed.

A multi-modal approach, including advanced imaging techniques and biomarkers, is crucial for diagnosing and characterizing CA, as the basis to guide appropriate treatment strategies.

## Therapy

**Fig. 2:**
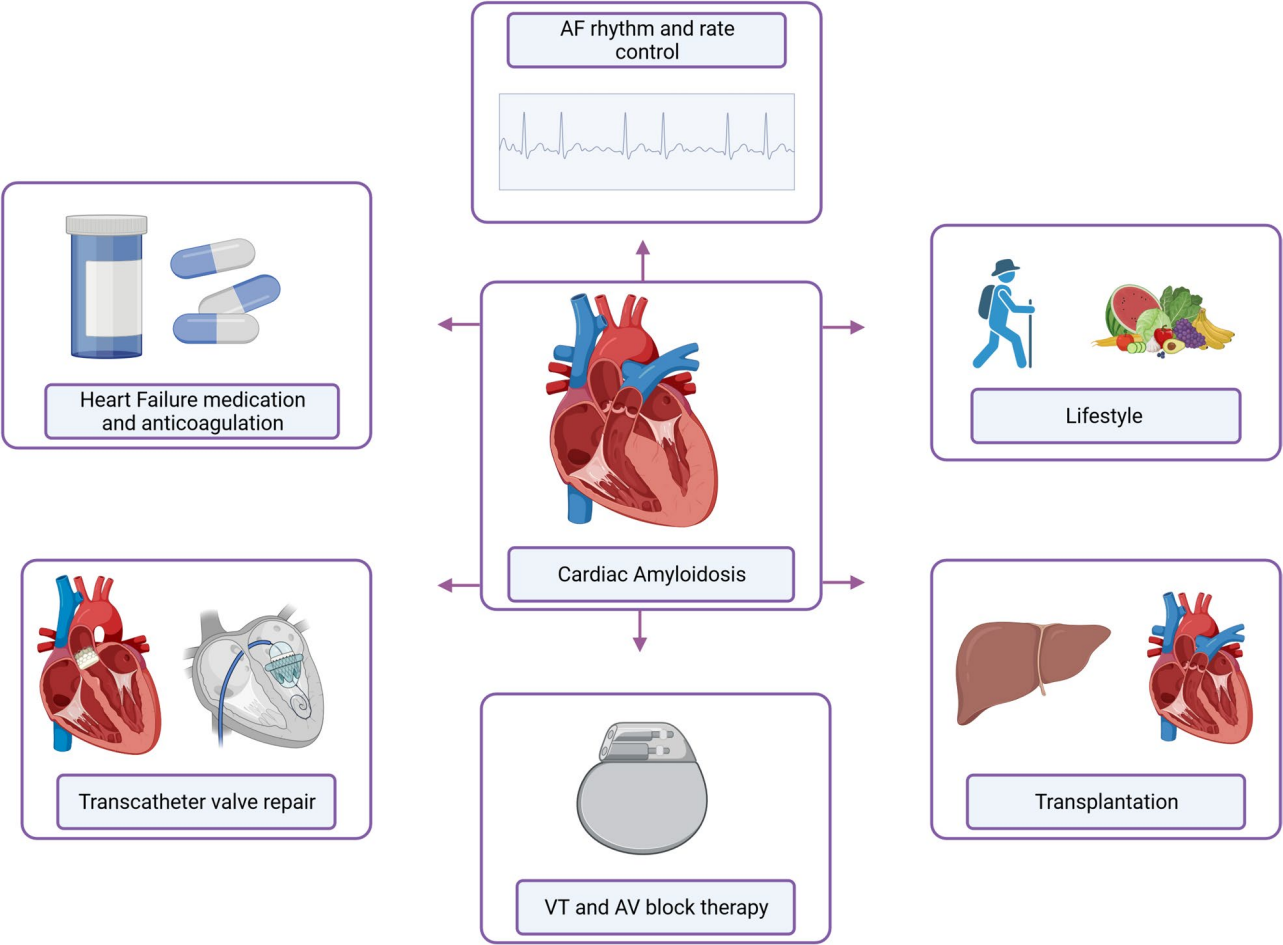
Symptomatic therapy for patients with cardiac amyloidosis (AF = atrial fibrillation, AV = atrio-ventricular, VT = ventricular tachycardia)

While AL-amyloidosis is treated with anti-plasma cell therapies since the 1960s [21], ATTR-amyloidosis was only treated symptomatically for a long time due to a lack of therapeutic options. In recent years more targeted therapeutic approaches and improvements have been developed, which will be discussed in this review (see Table 1). A literature search on clinicaltrials.gov revealed that more than 100 clinical trials for new therapies for amyloidosis are currently ongoing (as of 01 Dec 2023), which underlines the high relevance of the disease. Treatment of CA depends on the type (mostly ATTR versus AL) and clinical symptoms. Figure 2 shows an overview for symptomatic therapy for patients with CA.

**Table 1:** Study Overview Cardiac Amyloidosis

Reference	Drug	Dose (adminis-tration)	Company	Study name (year)	Design	Population (size)	Pathway	Outcomes	Cost per patient and year
ATTR									
Stabilizer									
Maurer et al.	Tafamidis	80mg/day and 20mg/day (orally)	Pfizer	ATTR-ACT (2018)	multicenter, international, double-blind, placebo-controlled phase 3	ATTR-cardiomyopathy (*n*=441)	TTR-Stabilizer	Reduction of all-cause mortailty and cardiovascular-related hospitalization	~157,000 Euro
Siddiqi et al.	Diflunisal	250mg/twice a day (orally)	-	- (2022)	retrospective cohort study	ATTR-cardiomyopathy (*n*=104)	TTR-Stabilizer	improved survival and overall stability in clinical and echocardiographic markers	~240 Euro
NCT03860935	AG10	800mg/twice a day (orally)	BridgeBio Pharma (USA)	ATTRibute-CM	randomized, double-blind, placebo-controlled phase 3	ATTR-cardiomyopathy (*n*=632)	TTR-Stabilizer	overall win ratio favoring acoramidis in all-cause mortality, cardiovascular-related hospitalization, change from baseline NT-pro BNP + 6-minute walk distance	-
aus dem Siepen et al.	Epigallocatechin-3-gallate	600mg/day (orally)	-	- (2015)	observational	ATTR-cardiomyopathy (*n*=25)	TTR-Stabilizer	Decrease of LV myocardial mass (6%) in cMRI	~900 Euro
Silencer									
Benson et al.	Inotersen	284mg/once a week (subcutaneous)	Ionis Pharmaceuticals (USA)	NEURO-TTR (2018)	international, randomized, double-blind, placebo controlles phase 3	ATTR-polyneuropathie +/-cardiomyopathy (*n*=173)	Antisense oligonucleotid	Increase of Quailty of life, no significant changes in echocardiographic parameter	between 340,000 and 677,000 Euro
Coelho et al.	Eplontersen	45mg/once a month (subcutaneous)	Ionis Pharmaceuticals (USA)	NEURO-TTRansform (2019)	international, open-label phase 3	ATTR-polyneuropathie +/-cardiomyopathy (*n*=66)	Antisense oligonucleotid	Ongoing study	-
NCT04136171	Eplontersen	45mg/once a month (subcutaneous)	Ionis Pharmaceuticals (USA)	CARDIO-TTRansform (2023)	international, double-blind, randomized, placebo-controlled phase 3	ATTR-cardiomyopatahy (*n*>1400)	Antisense oligonucleotid	Ongoing study	-
Antibodies									
NCT04360434	NI006	0,3 to 60mg/once a month (iv)	Neuroimmune AG (Switzerland)	- (2023)	randomized, placebo-controlled, double-blind, dose escalation phase 1	ATTR-cardiomyopathy (*n*=40)	Monoclonal antibody	Reduction of cardiac tracer uptake on scintigraphy, reduced NT-pro BNP and troponin	-
NCT03336580	PRX004	0.1, 0.3,1,3,10 and 30mg/kg / once a month (iv)	Prothena Bioscience Limited (USA)	- (2020)	open-label, dose escalation phase 1	ATTR-cardiomyopathy (*n*=7)	Monoclonal antibody	Acceptable safety profile, enhancement of global longitudinal strain	-
Amyloid fibril disruption									
NCT03481972	Doxycycline / Tauroursodeoxycholic + standard supportive therapy	250mg/day Doxy + 750mg/day TUDCA (orally)	-	- (2023)	randomized phase III	ATTR-cardiomyopathy (*n*=102)	Disruption (antibiotic + hydrophilic bile acid)	Ongoing study	~ 1160 Euro
AL									
Chemotherapy + Antibodies									
Kastritis et al.	Daratumumab (+ bortezomib, cyclophosphamide, dexamethason (CyBorD))	1800mg/weekly (week 1-2), 1800mg/every two weeks (week 3-6), 1800mg/every 4 weeks (from week 7) (subcutaneous)	Janssen Pharmaceutica (Belgium)	ANDROMEDA (2021)	international, open-label, randomized, active-controlled phase 3	AL amyloidosis (*n*=388)	Monoclonal antibody	Addition of Daratumumab higher frequencies of complete response and survival free from major organ deterioration	~179,370 Euro
Gertz et al.	Birtamimab + SoC	24mg/kg/day + SoC every 28days (iv)	Prothena Therapeutics Ltd (Ireland)	VITAL (2019)	multicenter, global, double-blind, placebo-controlled phase 3	AL-amyloidosis (*n*=260)	Monoclonal antibody	No decrease in all-cause death for Mayo I-III	-
Gertz et al.	Birtamimab + SoC	24mg/kg/day + SoC every 28days (iv)	Prothena Therapeutics Ltd (Ireland)	AFFIRM-AL (2023)	multicenter, global, double-blind, placebo-controlled phase 3	AL-amyloidosis (*n*=140)	Monoclonal antibody	Improvement of QoL, improved cardiac functioning (6MWT) in Mayo stage IV	-
NCT04512235	CAEL-101 + SoC	- (iv)	Alexion Pharmaceuticals (USA)	-2025	double-blind, randomized, multicenter, international phase 3	AL-amyloidosis, Mayo IIIb with cardiac involvment (*n*=267)	Monoclonal antibody	Ongoing Study	-
NCT04504825	CAEL-101 + SoC	- (iv)	Alexion Pharmaceuticals (USA)	- (2024)	double-blind, randomized, multicenter, international phase 3	AL-amyloidosis, Mayo IIIb with cardiac involvment (*n*=124)	Monoclonal antibody	Ongoing Study	-
Antibodies									
NCT04131309	Monotherapy Daratumumab	16 mg/kg infusion (iv) or 1800 mg injection (sc) weekly for Cycles 1-2, every 2 weeks for Cycles 3-6 and every 4 weeks thereafter	Janssen Pharmaceutica (Belgium)	-	open-label, multicenter phase 2	AL amyloidosis (*n*=40)	Monoclonal antibody + chemotherapy	Ongoing Study	~179,370 Euro
NCT05201911	AT-03	- (iv)	Attralus Inc (USA)	- (2022)	single-arm, single dose phase 1	Systemic amyloidosis (*n*=13)	Monoclonal antibody	Ongoing Study	-
NCT05521022	AT-02	- (iv)	Attralus Inc (USA)	- (2024)	single- and multiple-ascending dose escalation study in healthy volunteers and patients with systemic amyloidosis	Systemic amyloidosis (*n*=100)	Monoclonal antiboy	Ongoing Study	-
Amyloid fibril disruption									
Shen et al.	Doxycycline + CyBorD	200mg/day (orally) + 9 cycles CyBorD	-	- (2022)	multicenter, open-label, randomized controlled	AL amyloidosis (*n*=140)	Disruption (antibiotic + chemotherapy)	No prolongation of progression-free survival compared to CyBorD therapy alone	~200 Euro

*AL* light chain amyloidosis, *ATTR* transthyretin amyloidosis, *NT-pro BNP* N-Terminal pro-Brain Natriuretic Peptide

### Managing Cardiac Amyloidosis: General Principles

#### Heart Failure Medication

Treatment of heart failure symptoms like dyspnea, edema and syncope are often challenging in patients with CA. Apart from diuretics and mineralocorticoid receptor antagonists (MRA), standard of care (SoC) therapy is often not well tolerated in this patient group [22], mostly because of hypotension and chronotropic incompetence. Retrospective studies even showed that patients with CA receiving SoC, especially angiotensin-converting enzyme inhibitor (ACEi) and angiotensin II receptor blockers (ARBs) had more severe cardiac disease, because of vasodilatation and the inability to increase stroke volume [23•], due to the disease itself and the effect of ACEi and ARBs [24]. Low-dose betablockers were associated with a lower risk of mortality in patients with a left ventricular ejection fraction (LVEF) ≤40%. In patients with LVEF >40% there was no significant difference in survival [23•].

#### Rhythm Therapy

Extracellular amyloid infiltration leads to further abnormalities including arrhythmias with various presentations. Atrial fibrillation (AF) is very common in CA (prevalence varies between 15% and 40%) [25, 26]. Rate and rhythm control is essential in management of AF in this patient group. Low-dose betablockers and amiodarone appear to be well tolerated, while the available evidence for catheter ablation in these patients is poor with high rates of AF recurrence compared to non-CA patients [27]. Conduction abnormalities such as atrioventricular (AV) block are frequently associated with CA resulting in pacemaker implantation [28]. Furthermore, ventricular arrhythmias with a risk of sudden cardiac death exists especially in CA-patients, indication for ICD implantation remains undefined and there is a need for further large studies to elucidate the risk-benefit ratio of ICD implantation, particularly in presence of manifest multiple myeloma in AL-amyloidosis [29].

#### Anticoagulation

Patients with CA exhibit higher incidence of intracardiac thrombi, even in absence of rhythm disorders like AF [30]. A retrospective study with 100 ATTR-amyloidosis patients showed that 30 patients developed a thrombus in the left atrial appendage (LAA), 26 of them were on systemic anticoagulation (87%), indicating that the recommendation for anticoagulation based on CHA2DS2-VASc-Score may not be applicable for CA [31]. Hence, available position papers recommend to consider anticoagulation for all patients with CA and AF irrespective of the CHA2DS2-VASc-Score [2•, 32]. The use of novel oral anticoagulants compared to vitamin-k-antagonists was assessed in a retrospective trial including 217 ATTR-amyloidosis patients with AF compared to 73 non-CA patients with AF. No differences in thrombotic events and major bleedings were reported in CA [33]. Further prospective studies with larger cohorts are needed to target this important aspect of CA treatment.

#### Transplantation

Heart transplantation for patients with CA have to be carefully weighed against the potential risk of recurrence due to the systemic underlying cause of the disease [34]. Heart transplantation for ATTR-amyloidosis was first reported in 2003, with combined liver transplantation and good outcome (*n*=3, 1-year survival 66,7%, one patient died due to multiorgan failure 2 months after transplantation) [35]. A retrospective study with 14 ATTR-patients who underwent heart transplantation showed no amyloid recurrence in the transplant with a 5-year survival of 90% [36].

In 1984 the first reported AL-patient was heart transplanted who survived 10 years [37]. Due to a result of the negative effects of chemotherapy on heart failure symptoms a study suggested that patients with AL-amyloidosis should be transplanted before specific chemotherapy and stem cell support [38], but this was before usage of monoclonal antibodies. Patients with multiple myeloma are not eligible for heart transplantation [39].

Liver transplantation was the first treatment for ATTRh-amyloidosis, because the liver is the production source for TTR-tetramers. Currently, with new pharmacological treatments, liver transplantation is reserved for patients with relevant specific comorbidities and is considered rarely [40].

#### Interventions

The accumulation of amyloid fibrils in the heart valve is associated with thickened leaflets, and decreased orifice area, resulting in valve diseases like aortic stenosis (AS), mitral regurgitation (MR) and tricuspid regurgitation (TR) [41]. Patients with CA are frequently old and multimorbid and have a prohibitive surgical risk. A minimally invasive procedure for valve repair is therefore a favorable approach in the majority of these patients [41]. Transcatheter aortic valve replacement (TAVR) has been proven to be an effective and safe procedure in CA patients compared to non-CA patients [42]. Transcatheter edge-to-edge repair (TEER) for MR and TR is a well-established therapy for patients at high surgical risk with good results, particularly in HF patients with secondary mitral regurgitation if technically feasible [43–45]. Initial studies on the safety and efficacy of TEER in CA patients have already been completed [46], but the specific anatomy and conditions of CA patients makes the intervention particularly difficult and requires further investigations. Interventional valve procedures in CA patients should primarily be performed in experienced high-volume centers with a dedicated specialization on CA.

#### Nonpharmacological Treatment

Psychological support for patients with amyloidosis should be considered with the aim to relieve symptoms and to improve quality of life (QoL), considering that the prevalence of anxiety is 3-fold and of depression 4-fold higher in CA patients compared to the general population [47]. The progression of cardiac symptoms is accompanied by a reduction in QoL which can lead to social isolation [48]. Early psychotherapy programs and coping strategies are therefore beneficial. Apart from regular physical activity, a diet low in salt and high in vitamins is recommended for all patients with heart failure [22] to increase QoL and relieve symptoms.

#### Specific Therapy: ATTR-Amyloidosis

**Fig. 3:**
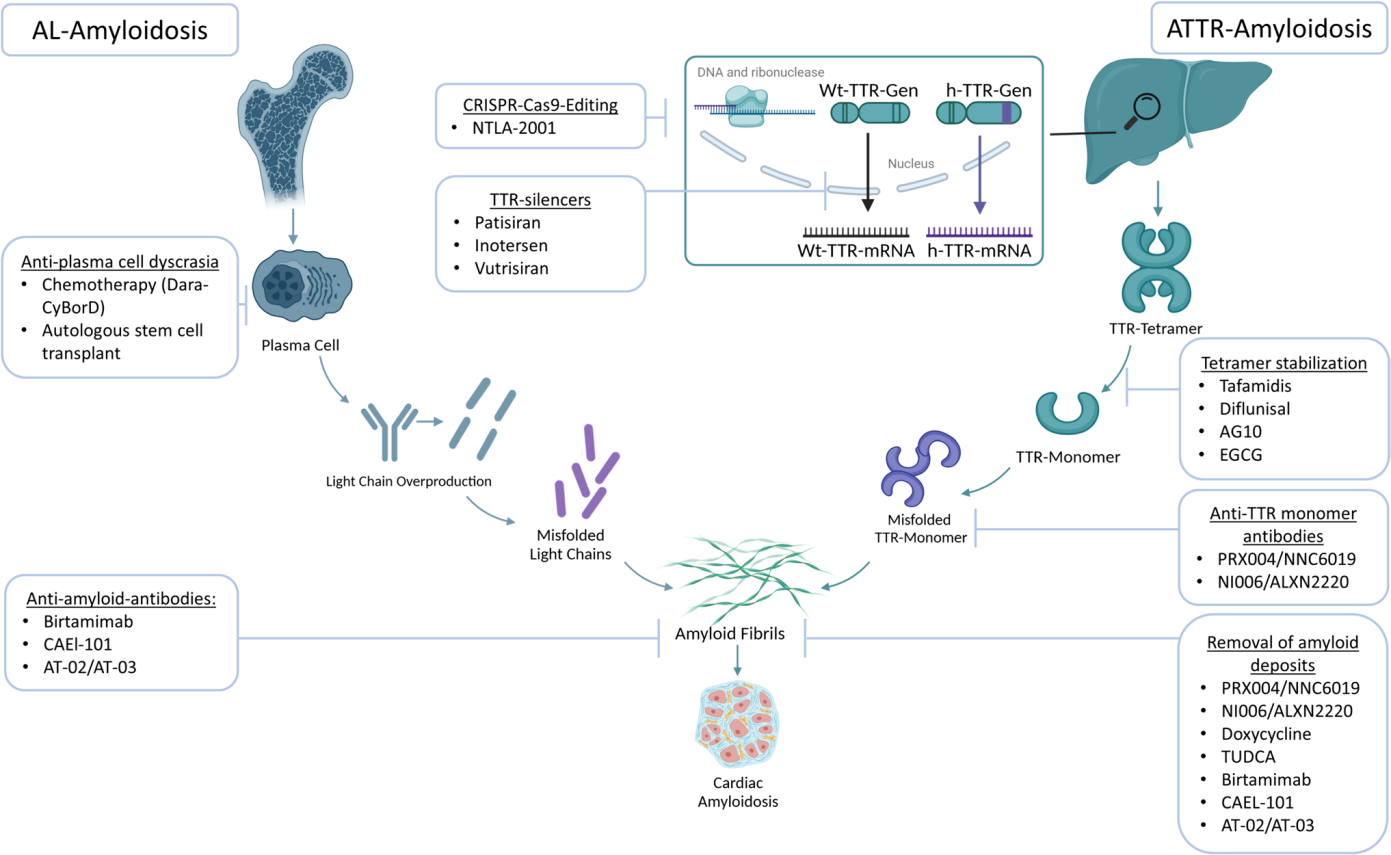
Targets and mechanisms of therapy for ATTR-amyloidosis (Cas = CRISPR-associated, CRISPR = clustered regularly interspaced short palindromic repeats, Dara-CyrBorD = daratumumab/cyclophosphamid/bortezomin/dexamethason, h = hereditary, mRNA = messenger RNA, TTR = transthyretin, Wt = wild-type)

Transthyretin (TTR) is a tetramer composed of four subunits produced in the liver. The tetrameric structure binds and transports thyroxine (T_4_) [49]. When TTR misfolds or dissociates into monomers, it becomes more prone to misfolding leading to amyloid formation [50]. There are different therapeutic approaches for the treatment of ATTR-amyloidosis that target different signaling pathways (see Fig. 3).

#### Stabilizers

One of the key therapeutic approaches for ATTR-amyloidosis is the use of stabilizers. The primary aim of TTR stabilizers is to maintain the TTR protein in its natural tetrameric structure to reduce TTR monomers that are prone to amyloid formation. Stabilizers work by binding to the TTR protein, preventing its misfolding or dissociation into monomers, which slows down the progression of ATTR-amyloidosis [7•, 51]. This binding can be reversible or irreversible, depending on the specific stabilizer helping to prevent further amyloid deposition.

Tafamidis, which binds to the thyroxine-binding site of the TTR tetramer and prevents its dissociation into monomers [7•], was approved by the FDA in 2019 and by the EMA in 2020 for treating ATTRwt-CA and ATTRh-CA. At present, Tafamidis is the only approved drug for cardiac ATTR-amyloidosis treatment. The ATTR-ACT trial showed that Tafamidis is associated with doubling of life expectancy compared to SoC therapy [52]. Compared to 177 patients who received placebo, the 264 patients who received Tafamidis showed a lower all-cause mortality (42.9% vs. 29.5%) and a lower rate of cardiovascular-related hospitalizations [7•]. Acoramidis is another potential ATTR-stabilizer which is being studied in clinical trials. It mimics the structural influence of the protective T119M mutation, which reduces the dissociation rate of tetrameric TTR compared to wild-type TTR [53]. The ATTRibute-CM trial, a multinational, randomized, double-blind, placebo-controlled phase 3 trial, investigated the effect of Acoramidis in CA. The study demonstrated a reduction in all-cause mortality compared to placebo, with an absolute risk reduction of 6,4% [54].

Diflunisal, a nonsteroidal anti-inflammatory drug (NSAID), was evaluated as an experimental therapy for CA and by binding also to the T_4_-binding site of TTR and stabilizes the tetramer [55]. A few clinical studies showed a positive effect of Diflunisal in patients with CA by stabilization of left ventricular (LV) wall thickness and cardiac biomarkers [56, 57]. However, the side effects of cyclooxygenase (Cox)-1 and Cox-2 inhibition, such as gastrointestinal bleeding or deterioration of renal function, should be considered.

The green tea extract epigallocatechin-3-gallate (EGCG) binds to the TTR-tetramer. Unlike Tafamidis, which binds to the T_4_-binding site, EGCG binds to the surface of the protein and stabilizes the tetramer. Furthermore it has been shown that EGCG can destroy TTR fibrils in an *in vitro* model [58]. An observational study analyzed 25 CA patients who had consumed green tea extract (600mg EGCG per day). CMR showed that myocardial mass was reduced, but interestingly, echocardiographic parameters showed no changes [59].

#### Silencer

The use of genetically modified ribonucleic acid (RNA) as a therapeutic approach for diseases has been important not only since the announcement of the Nobel Prize in Medicine in 2023. Another pivotal avenue in managing ATTR-amyloidosis focuses on silencers, strategically designed to address the root cause of the condition by reducing the production of TTR-protein. They utilize RNA interference (RNAi) technology to target the messenger RNA (mRNA) responsible for producing TTR. There are two promising pathways for silencing mRNA: binding of anti-sense oligonucleotides (ASOs) like Inotersen and Eplontersen and binding of small interfering RNAs (siRNA) like Patisiran and Vutrisiran [60].

The double-stranded siRNA can be delivered into the cell, where it is introduced into the RNA-induced silencing complex (RISC). RISC contains siRNA and several proteins which initiates mRNA cleavage resulting in decreased production of TTR-protein [61]. Patisiran acts as an siRNA in hepatocytes and blocks the production of TTR-protein by inducing splitting of TTR-mRNA [62]. In the APOLLO-B trial, Patisiran was compared to control in patients with CA and showed a reduction in all-cause hospitalization, HF visits and death [63•], however, did not show any significant advantage over the established therapy. Another siRNA drug for the treatment of hATTR and wtATTR amyloidosis is currently being investigated in the HELIOS-B study (NCT04153149). In a phase I study vutrisiran has already shown that the TTR-protein concentration was significantly reduced and that no serious side effects occurred [64], and the results of HELIOS-B can be expected in 2024. Vutrisiran is already approved for hATTR-polyneuropathy.

The same pharmaceutical company has also investigated a third siRNA drug in a large multicenter phase III study. However, in the ENDEAVOUR study, Revusiran showed that 12.9% of patients (*n*=140) who received siRNA therapy died (compared to 3% in the placebo group, *n*=66) with no clear causative mechanism identified, resulting in the trial being stopped prematurely [65].

Antisense oligonucleotides (ASOs) consist of 16-20 nucleotides, which bind to the mRNA and lead to degradation through blocking ribosomes and endogenous ribonuclease (RNase) H1, which are essential for translation [66]. Preclinical studies showed that ASOs to TTR significantly inhibit TTR synthesis and by that reduce serum levels of the protein [67, 68]. Inotersen, which was already approved for treatment of ATTR-polyneuropathy, showed no significant changes in echocardiographic parameters in the NEURO-TTR trial, a randomized, double-blind, placebo-control phase III trial where 172 patients with ATTR-polyneuropathy with and without cardiomyopathy were examined [69]. Smaller studies already proved positive outcomes on left-ventricle mass (11.4% decrease) and exercise tolerability (16.2 meter increase in 6-minute walk test) [70]. Another promising ASO is under current investigation. Eplontersen is tested in patients with ATTR-cardiomyopathy with NYHA class I-III. There is an approximate 30- to 50-fold increase in potency compared to Inotersen in preclinical studies and TTR levels in healthy volunteers in a phase I study were reduced by a mean of 86.3% [71]. First phase III results will be expected in 2025 (NCT04136171).

NTLA-2001 is the first treatment candidate which interferes with the clustered regularly interspaced short palindromic repeats and associated Cas9 endonuclease (CRISP-Cas9) pathway. CRISP-Cas9 is a promising new approach and one of the most efficient genome manipulation techniques, which is increasingly being studied in cancer therapy [72]. Here, this pathway is also used for treatment of CA. In contrast to ASOs, a human-optimised mRNA molecule encoding the Streptococcus pyogenes Cas9 protein is used instead of a specific siRNA for the TTR protein. Once in the hepatocyte, the Cas9-RNA can produce the Cas9 endonuclease, which forms a complex with the siRNA. This complex enters the cell nucleus and can specifically target the DNA helix of the TTR gene, leading to the depletion of the DNA [73]. This potential drug was tested in 6 patients with hATTR and polyneuropathy with and without TTR-cardiomyopathy and showed a significant reduction of TTR-levels. Further studies are expected for this targeted treatment of cardiac amyloidosis [74•].

#### Antibodies

As we explore the therapeutic frontiers for ATTR-amyloidosis, the role of immunotherapies, particularly monoclonal antibodies (mAbs), is emerging as a promising avenue. Antibodies might reshape the landscape of treating ATTR-cardiomyopathy. In the context of CA, immunotherapies can be developed to target and remove amyloid deposits in the heart or modulate the immune response to the disease by stimulating macrophages and giant cell phagocytosis supporting recovery of organ function [75]. Currently there are two potential mABs for treatment of ATTR-cardiomyopathy under investigation. NI006 was tested in 40 patients with ATTR-cardiomyopathy in a phase I trial. There were no drug-related serious adverse events, NT-pro BNP and troponin T levels decreased upon therapy. Cardiac tracer uptake in scintigraphy as a marker of CA-load was reduced indicating potential therapeutic efficacy. [76]. Another mAB designed to target and clear misfolded TTR-monomers is PRX004. Due to the coronavirus disease 2019 pandemic a phase I study was terminated, but 7 included patients showed an increase in global longitudinal strain and significant improvement in neuropathic pain (NCT03336580).

#### Amyloid Fibril Disruption

Within the spectrum of treatments for ATTR-amyloidosis, addressing the formation and persistence of amyloid fibrils is a pivotal consideration. Disrupting these fibrils holds significant importance, particularly in the context of treating ATTR-cardiomyopathy where cardiac involvement poses substantial challenges. Doxycycline, a tetracycline antibiotic, and tauroursodeoxycholic acid (TUDCA), a biliary acid, were tested in a preclinical mouse model for familial amyloid polyneuropathy (FAP) and showed a significant decrease of TTR deposition and tissue markers [77]. These findings were applied to a small study with 53 patients with ATTR-cardiomyopathy and showed improvements in left ventricular global longitudinal strain (-12 to -17%) and reduction in troponin-T levels [78]. A large phase III trial is still ongoing to validate these findings and to assess the net benefit including the potential reciprocal cardiotoxic effects of doxorubicin (NCT03481972).

#### Specific Therapy: AL-Amyloidosisuseful, not only for diagnostic, but

B or plasma cell clones can secrete higher amounts of clonal free light chains. The amino acid sequence of the light chains, especially the variable region, differs from cell clone to cell clone. In a few cases, these are proteins that can be deposited as amyloid; in these cases, AL-amyloidosis develops [79]. Risk stratification for every AL patient is required to find the right strategy: age, blood biomarker (e.g. NT-proBNP, troponin) and symptoms (NYHA class, Eastern Cooperative Oncology Group (ECOG) grade) are used to decide if a patient is low, intermediate or high risk [80]. The Cardiac Mayo classifications from 2004 and 2012 were established to assess the severity of the disease (see Table 2) [82, 83]. A detailed treatment of different individual antineoplastic treatment schemes adapted to the underlying hematological disorder is beyond the scope of this review. Here, we focus on the general therapeutic options for the treatment of first diagnosed AL-amyloidosis and exclude therapy options for recurrence and non-responders.

**Table 2:** Mayo staging system based on serum biomarkers

Stage	Biomarker
I	TnI < 0,1 ng/mL + BNP < 81 pg/mL
II	TnI > 0,1 ng/mL or BNP > 81 pg/mL
IIIa	TnI > 0,1 ng/mL + BNP > 81 pg/mL
IIIb	TnI > 0,1 ng/mL + BNP > 7001 pg/mL

*BNP* B type natriuretic peptide, *TnI* troponin I. Adapted from Wechalekar et al. JACC CardioOncol 2022 [81]

#### Autologous Stem Cell Transplantation

Patients with low-risk after revised Mayo staging, estimated 10-20% of all AL-amyloidosis patients, NYHA-classification < III, age < 70 years and biomarkers in the normal range are suitable for high dose chemotherapy with melphalan following autologous stem cell transplantation (ASCT) [84]. A ten-year study showed that 43% of all patients who underwent melphalan treatment and ASCT survived [85]. 40% of patients with AL-amyloidosis even showed complete response after ASCT determined by hematological response criteria [86]. However, these data were derived before bortezomib- and daratumumab-containing therapies were used. The value of high-dose therapy with autologous blood stem cell transplantation has not yet been prospectively investigated. This therapy option is now only used in selected cases as part of first-line therapy [87].

### Anti Plasma Cell Therapy

The outcome of patients with AL-amyloidosis is strongly associated with the severity of organ involvement, especially the heart. Modified Mayo classification from 2004 is particularly important for the choice of therapy [81, 83]. In patients with Mayo 2004 stages I to IIIa, based on the data of the ANROMEDA-Study [88], a combination of Daratumumab with Cyclophosphamide, Bortezomib and Dexamethason is standard of care. In some cases, e.g. due to insufficient response to therapy or existing organ damage for which this combination cannot be given, other combinations, e.g. with Melphalan and Lenalidomide, are used [80]. Mayo stage IIIb patients have been excluded from clinical trials in the past, so no prospective data have been published. In a retrospective study examining 119 patients with Mayo stage IIIb from several centers, a clear benefit was shown by adding Daratumumab to the anti-plasma cell therapy [89]. Daratumumab as monotherapy for Mayo stage IIIb is currently tested in a phase II trial, first results are expected in 2025 (NCT04131309).

### Anti-Amyloid-Antibodies

Three antifibril antibodies are under investigation for treatment of cardiac AL-amyloidosis (Birtamimab, CAEL-101 and AT-02/03) [81]. Birtamimab, a mAB, binds to an epitope of immunglobuline (Ig) light chains, presumably inducing phagocytosis of amyloid-deposits by macrophages and neutrophiles [90]. Birtamimab was also shown to bind serum amyloid protein A (AA) deposits in a rare form of amyloidosis associated with chronic inflammation (e.g., autoimmune disease, familial Mediterranean fever). The VITAL trial showed no significant improvement for all-cause mortality in patients with cardiac involvement of AL-amyloidosis for Mayo 2012 stage I-IV [91]. *Post hoc* analyses revealed a survival advantage in the Mayo 2012 stage IV subgroup [92]. The effect of Birtamimab is currently being investigated prospectively and randomized in the AFFIRM-AL study.

When amyloid Ig light chains are misfolded, a cryptic epitope is exposed [93] where the chimeric mAB CAEL-101 is able to bind [94]. In phase I and II studies patients treated with CAEL-101 showed therapeutic response by improved serum biomarkers and imaging modalities [95]. Two phase III randomized trials with CAEL-101 are ongoing (NCT04504825, NCT04512235) in patients with Mayo 2004 stages IIIA and IIIB.

By binding to serum amyloid P (SAP) protein AT-02 and AT-03 are under current investigations (NCT05521022, NCT05201911). These mABs bind to different forms of amyloid proteins and support removal of amyloid deposits [96].

### Amyloid Fibril Disruption

Doxycycline and EGCG have already been used in ATTR-amyloidosis in the experimental setting as mentioned before (see ATTR-amyloidosis). Due to their non-specific activity against amyloid fibrils, they may exhibit a comparable distinct effect in AL-amyloidosis. A phase IV study compared Doxycycline therapy plus CyBorD with CyBorD therapy alone and showed no improvement pf progression free survival with Doxycycline on top of chemotherapy [97]. A randomized phase II/III trial comparing Doxycycline with standard supportive care in patients undergoing Bortezomib therapy is now active, and results are expected soon (NCT03474458). EGCG is also being tested for AL-amyloidosis in two phase II trials, and first results are awaited (NCT02015312 and NCT01511263).

## Conclusion

As diagnostics and awareness continue to improve, the prevalence of CA is increasing. The highly topical, dynamic research landscape drives many ongoing studies, triggered by the high prevalence and incidence of this disease. With upcoming approvals, treatment options are becoming more complex, which requires expertise and often multidisciplinary cooperation. For ATTR-amyloidosis, only tafamidis is currently available, but several other drugs are in late clinical testing, which will significantly improve the treatment options. Treatment of AL-amyloidosis includes approved anti-plasma-cell therapies with a large variety of highly potent hematologic treatment strategies. A close collaboration between the individual disciplines is paramount to diagnose and treat this complex disease according to current standards [2•].

**Fig. 4:**
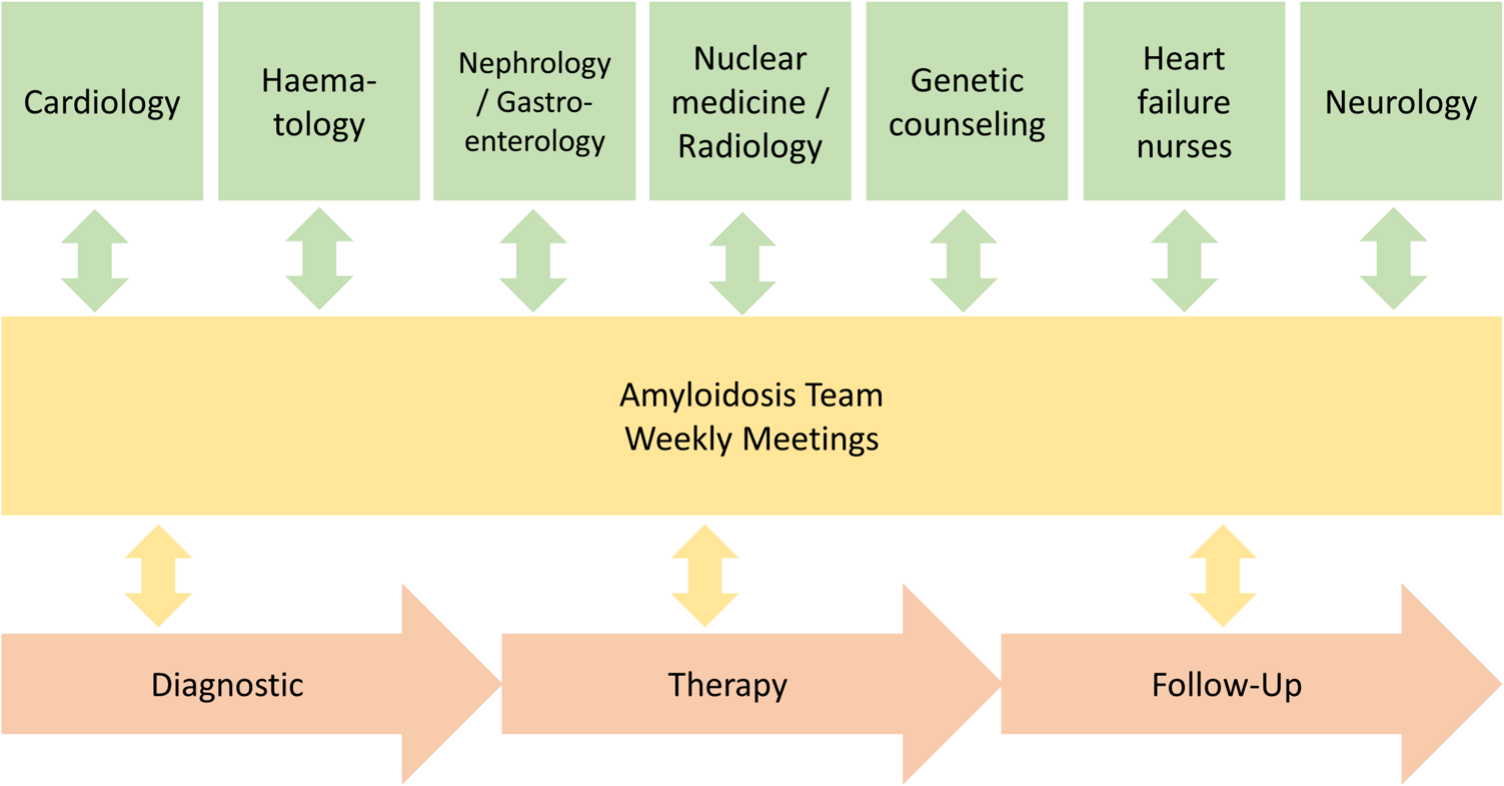
The amyloidosis team. Individual disciplines with diverse specialties are needed for a multidisciplinary approach, aiming to achieve best care and therapy of CA patients

If CA is suspected, patients should be referred to amyloidosis centers for advanced diagnostics and connection to advanced outpatient management [98]. An amyloidosis team with diverse specialties can help to coordinate tailored care and therapy and should be obligatory for amyloidosis centers. By implementing sub-disciplines, such as: cardiology, hematology, nephrology, gastroenterology, neurology, nuclear medicine, radiology, genetic counselling and heart failure nurses can provide best therapy for CA-patients (see Fig. 4) [3•]. Early diagnosis also enhanced through genetic testing for ATTR-amyloidosis, followed by genetic editing therapies can help to treat patients before developing symptoms. In the future, antifibril therapy for ATTR- and AL-amyloidosis will be hopefully safe, effective, and available for the broad majority of the growing population of CA patients. In addition to medical treatment, close patient care also plays a significant role, with amyloidosis centers and boards being particularly important.

## Data Availability

No datasets were generated or analysed during the current study.
